# Tocilizumab in the treatment of rapidly evolving COVID-19 pneumonia and multifaceted critical illness: A retrospective case series

**DOI:** 10.1016/j.amsu.2020.10.061

**Published:** 2020-11-05

**Authors:** Ahmed Mady, Waleed Aletreby, Basheer Abdulrahman, Mohammed Lhmdi, Alfateh M. Noor, Saleh A. Alqahtani, Ibrahim Soliman, Abdulrahman Alharthy, Dimitrios Karakitsos, Ziad A. Memish

**Affiliations:** a Critical Care Department, King Saud Medical City, Riyadh, Saudi Arabia; b Department of Anesthesiology and Critical Care, Tanta University Hospitals, Tanta, Egypt; c Department of Medicine, Johns Hopkins University, Baltimore, USA; d Department of Medicine, South Carolina University School of Medicine, Columbia, SC, USA; e Critical Care Department, Keck School of Medicine, University of Southern California, Los Angeles, CA, USA; f Research & Innovation Centre, King Saud Medical City, Riyadh, Saudi Arabia; g Hubert Department of Global Health, Rollins School of Public Health, Emory University, Atlanta, GA, USA; h College of Medicine, Alfaisal University, Riyadh, Saudi Arabia; i Department of Medicine, King Faisal Specialist Hospital & Research Center, Riyadh, Saudi Arabia

**Keywords:** COVID-19 pneumonia, Tocilizumab, Acute respiratory failure, Mechanical ventilation

## Abstract

**Background:**

COVID-19 associated critical illness characterized by rapidly evolving acute respiratory failure (ARF) can develop, especially on the grounds of hyperinflammation.

**Aim and methods:**

A case-series of 61 patients admitted to our intensive care unit (ICU) between August 12 and September 12, 2020 with confirmed COVID-19 pneumonia and rapidly evolving ARF requiring oxygen support therapy and/or mechanical ventilation was retrospectively analyzed. We examined whether intravenous administration of tocilizumab, a monoclonal interleukin-6 receptor antibody, was associated with improved outcome. All patients received empiric antivirals, dexamethasone 6 mg/day for 7 days, antibiotics, and prophylactic anticoagulation. Tocilizumab was administered at a dosage of 8 mg/kg [two consecutive intravenous infusions 12 h apart]. Outcome measures such as mortality on day-14, ICU length of stay, and rate of nosocomial acquired bacterial infections were also analyzed. Results: Patients were males (88.2%) aged 51 [interquartile range (IQR): 42.5–58.75)], with admission Acute Physiology and Chronic Health Evaluation (APACHE) 4 score of 53 (IQR: 37.75–72.5), and had more than one comorbidity (62.3%). On admission, twenty nine patients (47.5%) were mechanically ventilated, and thirty two patients (52.5%) were receiving oxygen therapy. No serious adverse effects due to tocilizumab therapy were recorded. However, twelve patients (19.6%) developed nosocomial acquired infections. ICU length of stay was 13 (IQR: 9–17) days, and mortality on day-14 was 24.6%. Six patients were shifted to other hospitals but were followed-up. The overall mortality on day-30 was 31.1%. Non-mechanically ventilated patients had higher survival rates compared to mechanically ventilated patients although results were not significant [hazards ratio = 2.6 (95% confidence intervals: 0.9–7.7), p = 0.08]. Tocilizumab did not affect the mortality of critically ill COVID-19 patients.

**Conclusion:**

Tocilizumab could be an adjunct safe therapy in rapidly evolving COVID-19 pneumonia and associated critical illness.

## Highlights

• Rapidly evolving respiratory failure is a hallmark of COVID-19-critical illness.
• Tocilizumab could be an adjunct safe therapy in critically ill COVID-19 patients.
• Tocilizumab did not affect the mortality of COVID-19 critically ill patients.
• The prevalence of bacterial infections post-tocilizumab therapy was 19.8%.

## 1 Introduction

Coronaviruses are enveloped RNA viruses of zoonotic pathogenicity that can cause severe respiratory disorders such as the Middle East Respiratory Syndrome (MERS) and the Severe Acute Respiratory Syndrome (SARS-CoV) [1,2]. The novel SARS-CoV-2 disease (COVID-19) emerged from China, in 2019, and spread worldwide [3,4]. COVID-19 was declared a pandemic by the World Health Organization (WHO) on March 11, 2020 [5]. To date, more than 41.550.000 infected cases were reported, while the death toll reached to 1.137.332 patients worldwide [6]. The clinical presentation of COVID-19 ranged from asymptomatic cases to critical illness requiring intensive care unit (ICU) admission [3,4,7,8]. Most COVID-19 cases were mild necessitating only minimal oxygen support during the acute phase of hospitalization [9]. COVID-19 associated critical illness was characterized by acute respiratory failure, sepsis-like features, thromboembolic disease, extra-pulmonary manifestations and multi-system organ failure [3,4,7,8].

Although the underlying pathophysiology of COVID-19 is extremely versatile [10,11], previous studies reported that critically ill patients had higher plasma concentrations of proinflammatory cytokines such as interleukin (IL)-6, IL-10, granulocyte colony stimulating factor, tumor necrosis factor amongst others [12]. Post-mortem lung tissue histopathology examinations revealed variable degrees of alveolar edema, proteinaceous exudate, inflammatory cellular infiltration, and microthrombosis [[13], [14], [15]]. Apart from the established role of COVID-19 associated severe thromboembolic disease [[16], [17], [18]], other cofactors such as dysregulated host immune response, and hyperinflammation could enhance the development of lung tissue damage and ensuing fibrosis [19,20]. However, only a subgroup of COVID-19 patient develops fulminant respiratory failure for reasons that remain obscure. The optimal management of these cases is an on-going challenge. IL-6 is a pivotal inflammatory mediator in the development of COVID-19 associated hyperinflammation; however, increased levels of other routine laboratory parameters such as C-reactive protein (C-RP), lactate dehydrogenase, ferritin, and D-dimers along with persistent lymphocytopenia were reported in COVID-19 patients with rapidly evolving respiratory failure [[6], [7], [8], [9], [10], [11], [12]]. Tocilizumab (TCZ), which is a recombinant, humanized monoclonal antibody against both soluble and membrane bound IL-6 receptors, was suggested to mitigate the COVID-19 associated cytokine storm [[21], [22], [23]], and was used in the management of severe COVID-19 but with variable clinical results [24,25]. In this retrospective, single center study, we present the clinical features of COVID-19 critically ill patients who underwent therapy with TCZ, and outline their course and outcome.

## 2 Methods

We retrospectively analyzed patients with severe COVID-19 who were admitted to our level III 300 bed ICU (King Saud Medical City, Riyadh, Saudi Arabia) between August 12 and September 12, 2020. Inclusion criteria were [26]: 1) Adult ≥ 18 years old, 2) ICU admission with at least one of the following indications: a) mechanical ventilation, b) respiratory rate > 30 b/min, c) peripheral oxygen saturation (SpO_2_) < 90% on room air, d) SpO_2_ to fraction of inspired oxygen ratio (FiO_2_) < 300, and 3) administration of tocilizumab. SARS-CoV-2 infection was confirmed by Real-Time-Polymerase-Chain-Reaction (RT-PCR) assays according to WHO recommendations [27], and using QuantiNova Probe RT-PCR kit (Qiagen) in a Light-Cycler 480 real-time PCR system (Roche, Basel, Switzerland) [28]. Exclusion criteria were: 1) pregnancy, 2) known immune suppression/deficiency status, 3) specific contraindications for the use of TCZ including suspected or confirmed bacterial infection, active diverticulitis or gastrointestinal tract perforation, neutropenia (0.500 × 10^3^ cells/uL) and thrombocytopenia (50 × 10^3^ cells/uL), and 3) two consecutive negative RT-PCR tests for SARS-CoV-2 taken at least 48 h apart. Our aim was to analyze the clinical course of critically ill COVID-19 patients who received TCZ. Outcome measures such as 14-day ICU survival, tocilizumab side-effects, and subgroup analysis between mechanically ventilated and non-mechanically ventilated COVID-19 patients were also reported. All enrolled patients were treated with empiric antiviral medications (lopinavir 400 mg + ritonavir 100 mg or ribavirin 400 mg every 12 h for 14 days), antibiotic prophylaxis (azithromycin, ceftriaxone or piperacillin/tazobactam for 14 days), dexamethasone 6 mg/day (for 7 days), and supportive care as per ICU protocol [29,30]. All patients received baseline weight- and renal function-adjusted doses of low-molecular-weight heparin thromboprophylaxis unless contraindicated (enoxaparin 20 mg once daily if < 50 kg; enoxaparin 40 mg once daily if 50–100 kg; 40 mg twice daily if 101–150 kg; 60 mg twice daily if > 150 kg). Also, intubated patients received ARDS-net and prone positioning ventilation, while non-intubated patients received oxygen support therapies via high flow nasal cannula (HFNC), helmet continuous positive airway pressure (H-CPAP), or Venturi masks [31]. Indication for TCZ was based on treating physician's evaluation for fulminant respiratory failure, which was refractory to the aforementioned therapies. TCZ (Actemra®, Roche Holding AG, Basel, Switzerland) was administered at a dosage of 8 mg/kg (max 800 mg) by two consecutive intravenous infusions 12 h apart. A third infusion, given 24 h apart from the second, was optional according to the clinical response. All epidemiologic and clinical data were retrieved by the patients' electronic medical records and were retrospectively analyzed. Putative side-effects of TCZ therapy during ICU hospitalization such as development of bacterial infections: Ventilator Associated Pneumonia (VAP; defined as respiratory culture positive or respiratory PCR plus compatible clinical, and radiological findings) [32], blood stream infection (BSI; one or more positive blood cultures associated with systemic signs of infection such as fevers, chills, and/or hypotension), and urinary tract infection (UTI; defined as one or more positive urinary cultures associated with systemic signs of infection) were also analyzed [33]. This case-series is compliant with the PROCESS guidelines as detailed elsewhere [34]. The study was conducted according to the principles of the Declaration of Helsinki and approved by our Institutional Review Board [35]. Written informed consent was obtained from patients (whenever possible) or their legal representatives.

## 3 Statistical analysis

Continuous variables are presented as medians with interquartile range (IQR), whereas categorical variables as numbers and/or percentages. Each variable was presented with its corresponding 95% confidence interval (CI). Subgroup analysis between mechanically ventilated and non-mechanically ventilated COVID-19 patients was performed by the student's t-test or the Wilcoxon rank sum test for continuous variables, and by the chi square test or the Fisher's exact test for categorical variables, as appropriate. Also, we tailored a cox proportional hazard model to compare the hazard of 14-day mortality in the aforementioned subgroups of patients. Results were presented as hazard ratio with log rank p value, and visually displayed as Kaplan Meier curve. The proportional hazard model was adjusted for all variables found to have significant difference in subgroup comparison, or deemed of clinical importance, and we used an enter method retaining variables with p value < 0.1. All statistical tests were two tailed, and considered significant with p value < 5%. Commercially available statistical package Stata® was used for the analysis (StataCorp. 2017. Stata Statistical Software: Release 15. College Station, TX: StataCorp LLC.).

## 4 Results

**Fig. 1:**
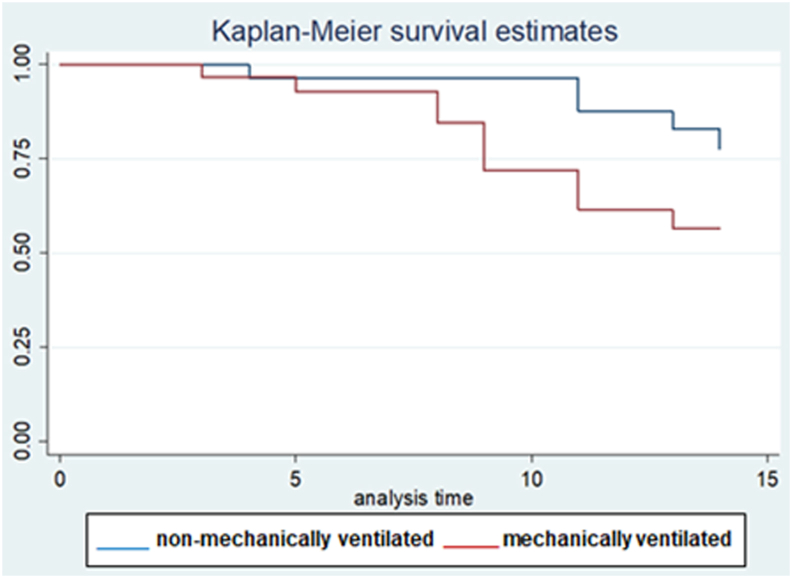
Survival on day-14 post admission for non-mechanically ventilated (n = 32), and mechanically ventilated (n = 29) COVID-19 patients that received tocilizumab [hazard ratio: 2.6, 95% confidence intervals: 0.9–7.7, p = 0.08].

Out of the eighty two patients that were admitted to our unit during the study period, sixty one consecutive COVID-19 patients fulfilled the inclusion criteria. The baseline characteristics of these sixty one patients who received TCZ are presented in Table 1. COVID-19 patient were mostly males (88.2%), aged 51 (IQR: 42.5–58.75) years old, and only a minority of them were of Middle Eastern origin. Forty five COVID-19 patients (73.7%) were of Asian origin. Upon ICU admission, their median Acute Physiology and Chronic Health Evaluation (APACHE) 4 score was 53 (IQR: 37.75–72.5); while the time period from symptoms onset to ICU admission was 5 (IQR: 3.75–6) days (Table 1). The majority of patients (62.3%) had more than one comorbidity. Usual comorbidities were diabetes mellitus (39.3%), arterial hypertension (21.7%), and obesity (24.6%). Upon inclusion, patients had lymphocytopenia and increased C-RP, and D-dimers. The prevalence of pulmonary embolism (PE) confirmed by computed chest tomography angiography was 11.4% in this series (Table 1). There were no cases of acute kidney injury necessitating continuous renal replacement therapy in this cohort. Also, no cardiovascular complications were observed during the study period. Upon admission, twenty nine patients (47.5%) were mechanically ventilated, and thirty two patients (52.5%) were non-mechanically ventilated but received oxygen support therapies [HFNC: twenty cases, H-CPAP: eight cases and Venturi mask: four cases]. ICU length of stay was 13 (IQR: 9–17) days, and hospital length of stay was 14 (IQR: 9–21) days. All patients received two doses of TCZ during the study period. No third dose was given. No other side effects of therapy (i.e., headache or gastrointestinal manifestations) were documented. During the study period, commonly prescribed antibiotics were: azithromycin (26 cases, 42.6%, for 5 days), piperacillin/tazobactam (18 cases, 29.5%, for 2 weeks), meropenem (13 cases, 21.3%, for 2 weeks), and vancomycin (13 cases, 21.3%, for 2 weeks). Twelve cases (19.6%) of nosocomial acquired infections during the study period were registered. VAP was identified in six mechanically ventilated cases [pathogens were: *Acinetobacter baumannii* (3 cases; 2 out 3 were multi-drug resistant infections sensitive to collistin), and *Pseudomonas species* (3 cases)]. BSI was recorded in five cases, three of which were central line associated BSI, [pathogens were: *Staphylococcus aureus*, *Vancomycin resistant enterococcus* (sensitive to tigecycline), and *Acinetobacter baumannii*]. UTI due to *Acinetobacter baumannii* was recorded in one case. TCZ therapy was not discontinued due to these infections as no episodes of septic shock or other serious adverse effects were documented. Moreover, these infections were not correlated with mortality. The clinical course of the sixty one COVID-19 patients who underwent TCZ therapy is illustrated in the charted Table 2. Six out of the sixty one patients were shifted to other health care facilities but remained under follow-up during the study period. Sixteen patients that were not mechanically ventilated (on admission) required eventually mechanical ventilation; while four mechanically ventilated patients were extubated and placed on oxygen support therapies. Mortality on day-14 post-ICU admission was (15 patients, 24.6%). Out of the six patients that were transferred to other hospitals one expired in the first fourteen days, while another four expired after a month of hospitalization; hence the overall mortality rate on day-30 post admission was (19 patients, 31.1%). RT-PCR for COVID-19 was negative, in survivors, approximately 17 (IQR: 13–21) days post-ICU admission. All survivors were followed-up for two weeks post-hospital discharge (out-patient clinic). No re-admissions and/or re-infections were observed in survivors. Subgroup analysis of mechanically ventilated versus non-mechanically ventilated patients revealed that the former had significantly higher body mass index and rate of hospital acquired infections, and increased ICU/hospital length of stay compared to the latter (Table 3). Survival on day-14 post-ICU admission was higher in the non-mechanically ventilated group of patients compared to the mechanically ventilated patients but did not reach statistical significance [hazard ratio: 2.6, 95% CI: 0.9–7.7, p = 0.08]. No other variables that entered in the cox proportional hazard model yielded a significant result. The administration of TCZ per se as an adjunct therapy did not have any effect on the mortality of critically ill COVID-19 patients. The survival model integrating the two subgroups of patients (mechanically ventilated versus non-mechanically ventilated) was well fitted (p value of chi^2^ = 0.009) and illustrated in Fig. 1 (Kaplan Meir curves).

**Table 1:** Baseline parameters of the sixty one COVID-19 critically ill patients who received tocilizumab.

Parameters	Median (IQR)/count (%)	95% CI
Age (years)	51 (42.5–58.75)	46–54.5
Gender (Male)	54 (88.2%)	77.4%–95%
Ethnicity (Middle Eastern)	16 (26.2%)	15.8%–39%
Onset of symptoms to ICU admission (days)	5 (3.75–6)	4–6
SpO_2_/FiO_2_ ratio	162 (145–209.2)	152–191.4
Hemoglobin (g/L, normal: 12–17)	13.2 (11.5–14.4)	11.9–13.7
White blood cells (cells/mm^3^, normal: 4–10)	8.27 (6.9–9.4)	7.1–9.1
Platelets (cells/mm^3^, normal: 150–450)	277 (187–353)	192–339
Lymphocyte count (10^9^/L, normal: 1.1–3.2)	0.8 (0.65–0.92)	0.72–0.88
C-reactive protein (mg/L, normal: 0–5)	31.7 (30.5–49.9)	34.8–42.7
Creatinine (mg/dL, normal: 0.6–1.2)	0.8 (0.6–1.1)	0.7–0.9
D-dimers (mcg/ml, normal: < 1)	2.4 (1.2–3.9)	1.7–3.1
Pulmonary embolism	7 (11.4%)	–
APACHE 4 score upon ICU admission	53 (37.75–72.5)	44.4–63.2
*Comorbidities*		
More than one comorbidity	38 (62.3%)	49%–74.4%
Diabetes mellitus	24 (39.3%)	27%–52.6%
Arterial hypertension	13 (21.7%)	12.2%–34.1%
Hypothyroidism	1 (1.6%)	0.04%–8.7%
Obesity (body mass index > 30)	15 (24.6%)	14.5%–37.3%
Active smoking status	37 (60.7%)	47.4%–73%
Mechanically ventilated	29 (47.5%)	–
Non-mechanically ventilated	32 (52.5%)	–
ICU length of stay (days)	13 (9–17)	11–15
Hospital length of stay (days)	14 (9–21)	11–19

Abbreviations: IQR = interquartile range, CI = confidence interval, ICU = intensive care unit, SpO2/FiO2 ratio APACHE 4 score = Acute Physiology and Chronic Health Evaluation score.

**Table 2:**
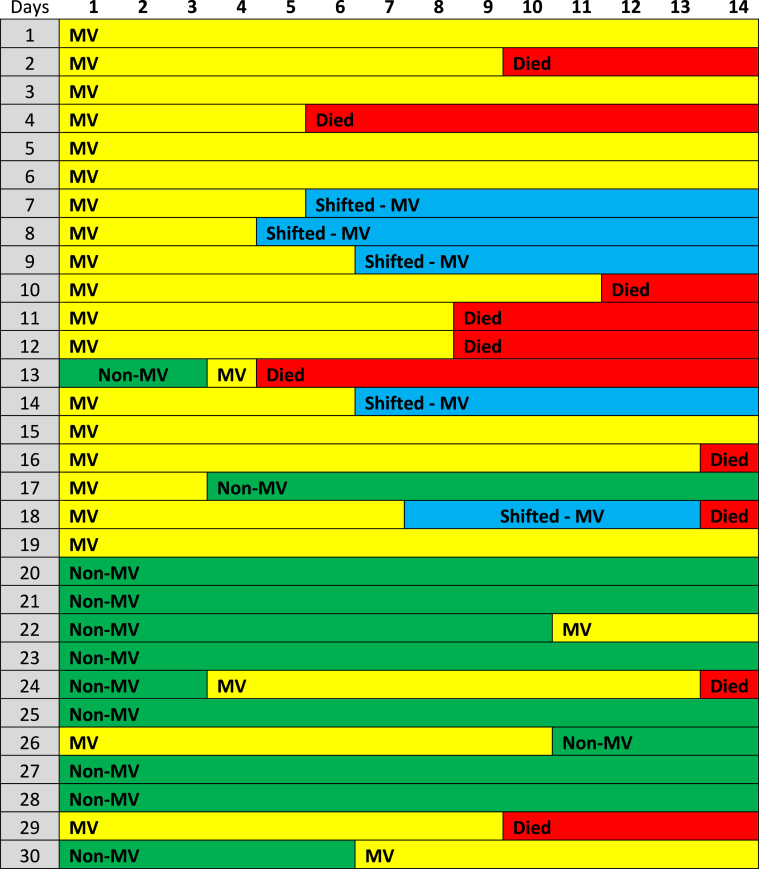

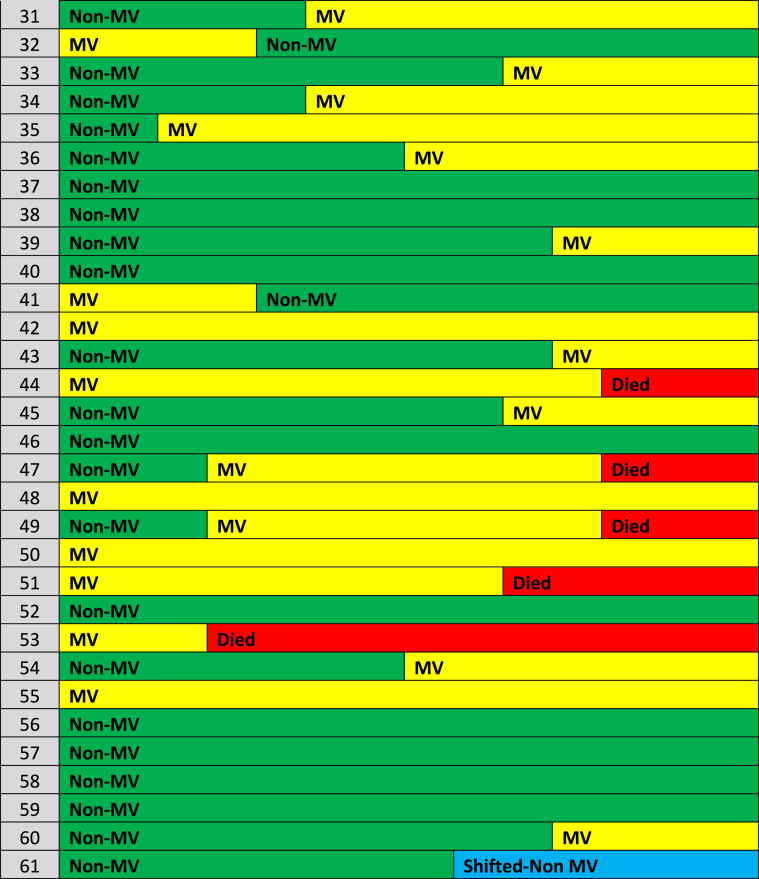
Summary clinical course chart* of the sixty one enrolled COVID-19 cases during the first fourteen days post admission.

Abbreviations: MV = mechanically ventilated, Non-MV = non-mechanically ventilated; * Green color: Non-mechanically ventilated patients, Yellow color: mechanically ventilated, Red color: expired on day-14 post admission, Blue color: shifted to other health care facilities but followed-up.

**Table 3:** Subgroup analysis between non-mechanically ventilated and mechanically ventilated COVID-19 patients who received tocilizumab.

Parameters	Non-mechanically ventilated (n = 32)	Mechanically ventilated (n = 29)	P value
Continuous variables (median (IQR)			
Age (years)	49 (44–61)	52 (42–57)	0.95
Hemoglobin (g/L)	13.4 (11.5–14.2)	12.9 (11.4–14.3)	0.82
White blood cells (cells/mm^3^)	8.25 (6.79–9.41)	8.32 (6.75–9.5)	0.64
Platelets (cells/mm^3^)	277.4 (186–358.5)	278.6 (187–354.9)	0.77
Creatinine (mg/L)	0.75 (0.5–0.9)	0.8 (0.5–1.1)	0.08
C-reactive protein (mg/L)	30.9 (24–47.6)	31.3 (30.5–47.5)	0.14
Onset of symptoms to ICU admission	5 (3–6)	5 (4–7)	0.87
ICU length of stay	10 (8–16)	14 (11–20)	0.04*
Hospital length of stay	11 (8–17)	16 (13–26	0.01*
APACHE 4 score	50 (36.2–69.4)	56 (38–72)	0.35
**Categorical variables (number, %)**			
Males	27 (84.4%)	27 (93.1%)	0.43
Survival on day-14 post admission	27 (84.4%)	19 (65.5%)	0.088
Obesity (body mass index >30)	3 (9.4%)	12 (41.4%)	0.018**
More than one comorbidity	19 (59.4%)	19 (65.5%)	0.62
More than one hospital acquired infection	3 (4.9%)	9 (14.7%)	0.01**

Abbreviations: IQR = interquartile range, ICU = intensive care unit, APACHE 4 score = Acute physiology and chronic health evaluation 4 score *Comparisons with Wilcoxon rank sum test or student's t-test as appropriate; p values < 0.05 were statistically significant; ** Comparisons with Fisher's exact test or chi^2^ test as appropriate, p values < 0.05 were statistically significant.

## 5 Discussion

The results of this retrospective, open-label, single-arm, single-center study can be summarized as follows. Unlike other similar studies from the Middle East region [21], our cohort consisted mainly of Asian males with a median age of 51 years old. Our reported mortality rates of 24.6% (on day-14 post-ICU admission) and 31.1% (on day-30 integrating patients that were shifted to other hospitals) are comparable with recently published studies [36]. Lower mortality rates ranging from 7% to 12% were previously reported by other authors, although the percentage of non-mechanically ventilated and presumably less critically ill patients enrolled in these studies was significantly higher [21,37]. In contrast, a large multicenter study integrating a longer follow-up period reported mortality rates post TCZ therapy up to 49%, which is in accordance with our findings [38]. Several cofounders such as comorbidities, thromboembolic disease, and mechanical ventilation affect mortality in COVID-19 studies [3,4,7,8]. The incidence of PE in our series was relatively low; however all patients received prophylactic anticoagulation based on a standardized protocol [[16], [17], [18]]. Also, the majority of our patients suffered from more than one comorbidity with diabetes mellitus and arterial hypertension being the most commonly observed [3,4]. We failed to show any differences in mortality between mechanically ventilated versus non-mechanically ventilated patients [3,4,7,8] but our retrospective study was significantly underpowered. However, subgroup analysis revealed higher body mass index and rate of nosocomial acquired infections in the COVID-19 mechanically ventilated subgroup of patients versus the non-mechanically ventilated group, which is in accordance with previous reports [[39], [40], [41], [42], [43], [44], [45], [46], [47]]. In our study, the incidence of nosocomial acquired infections based on strict clinical criteria was 19.6%, which although an important cofactor did not affect mortality during TCZ therapy. Other studies reported rates of positive cultures from various sites up to 54% during TCZ treatment although nosocomial acquired infections were not clearly defined and/or evaluated [47]. Surely, the immunomodulatory effect of TCZ, especially in mechanically ventilated patients, and thus the putative infection risk cannot be underestimated; however the administration of the medication in a controlled ICU environment appears to be relatively safe. The aforementioned concerns remain to be further clarified in upcoming TCZ randomized control trials [48,49]. Recently, a therapeutic regime involving a course of high-dose methylprednisolone, followed by TCZ if needed, showed that it may accelerate respiratory recovery, reduce hospital mortality and the rate of invasive mechanical ventilation in COVID-19-associated cytokine storm syndrome [50]. Of note, the preliminary findings of a phase III global TCZ versus placebo study (EMPACTA) showed that the administration of TCZ in patients with COVID-19 associated pneumonia plus standard of care were 44% less likely to progress to mechanical ventilation or death compared to patients who received placebo plus standard of care [(log-rank p-value = 0.0348; hazard ratio (95% CI) = 0.56 (0.32, 0.97)] [51]. The prevalence of bacterial infections that were observed in the EMPACTA study was comparable with our present results. Another point is that there is no consensus for the administration of TCZ in critically ill COVID-19 patients. Although a full panel of laboratory parameters was not available in this series, we found that most patients on admission had lymphocytopenia and increased levels of C-RP and D-dimers, indicating thus putative hyperinflammation [[21], [22], [23], [24], [25]]. This was a major study limitation as we did not establish a full blown cytokine storm [10,11,19], and thus TCZ was administered mainly on clinical grounds and the rapid progression of COVID-19 pneumonia [21,37,38]. Follow-up was also based on monitoring the evolution of the clinical picture, and thus the trend of possible changes in the laboratory parameters was not included in this study. Whether the occurrence of a cytokine release syndrome integrating elevated IL-6 levels should be a prerequisite for the administration of TCZ in COVID-19 patients remains to be fully elucidated in larger future studies. However, the presence of cytokine storm, which could facilitate the development of thromboinflammation and multi-system organ failure, could be a pivotal underlying mechanism in multifaceted COVID-19 critical illness [[10], [11], [12], [13], [14], [15], [16], [17], [18], [19], [20]]. Notably, during the pandemic, many ICU beds and ventilators may not be available at all times as was the case in our study. In that sense, our preliminary results showed that a subset of COVID-19 patients had a fast (one to two weeks) and sustainable response to TCZ therapy, which consequently facilitated the extubation of several cases and their hospital discharge (Table 2). Moreover, the observed mortality rate remained within the range of previously published COVID-19 studies despite the fact that all enrolled patients were critically ill. However, the administration of TCZ per se as an adjunct therapy did not have any effect on mortality of COVID-19 patients, which duplicates a recently published trial [52]. This retrospective case-series has several limitations, which prevent its generalizability. First, our patients might have improve to concomitant empiric antiviral and steroid therapy [4,[7], [8], [9], [10],^30^,^47^,^50^]. However, given the severity of their clinical status, the administration of TCZ might have helped by mitigating a full blown picture of hyperinfammation. Surely, the administration of steroids could have affected both the immune response and the viral clearance. The natural course of SARS-CoV-2's viremia is not well established, while reinfections and persistently positive RT-PCR results were reported [[53], [54], [55], [56]]. We are uncertain of the putative effect of TCZ on viral clearance although the RT-PCR results were negative in survivors post therapy. Surely, future studies should explore the indications and the optimal TCZ regime in COVID-19 critically ill patients [[48], [49], [50], [51], [52]]. In this study, the lack of a comparative control arm, the small number of enrolled patients, and the absence of a detailed laboratory documentation and follow-up rendered its power. Despite the aforementioned limitations, we showed that the administration of TCZ is a putative safe, adjunct therapy in COVID-19 critically ill patients.

## 6 Conclusion

The current results are preliminary and underpowered, and thus they should be interpreted accordingly. TCZ efficacy in severe COVID-19 needs to be validated by future randomized control trials. However, our data showed that the administration of TCZ as an adjunct therapy could be an alternative management option in rapidly evolving COVID-19 pneumonia and associated critical illness.

## Sources of funding

No funding was received for this study.

## Ethical approval

The study was approved by the Institutional Review Board of King Saud Medical City, Riyadh, Kingdom of Saudi Arabia [H-01-R-053, IORG0010374, H1RI-11-20-02].

## Consent

Written informed consent to participate in this study was obtained from the patients (whenever possible) or their legal representatives.

Written informed consent was obtained from the patient for publication of this case report and accompanying images. A copy of the written consent is available for review by the Editor-in-Chief of this journal on request.

## Author contribution

Ahmed Mady: study design, data collection/analysis, writing the original manuscript.

Waleed Aletreby: study design, data collection/analysis, writing the original manuscript.

Basheer Abdulrahman: study design, data collection/analysis, writing the original manuscript.

Mohammed Lhmdi: study design, data collection/analysis, writing the original manuscript.

Alfateh Noor: study design, data collection/analysis, writing the original manuscript.

Ibrahim Soliman: study design, data collection/analysis, writing the original manuscript.

Abdulrahman Alharthy: study design, data collection/analysis, writing the original manuscript.

Saleh A Alqahtani: data interpretation, supervision of the study, writing/editing the final version of the manuscript.

Dimitrios Karakitsos: data interpretation, supervision of the study, writing/editing the final version of the manuscript.

Ziad A Memish: data interpretation, supervision of the study, writing/editing the final version of the manuscript.

All authors reviewed the final version of the manuscript and agree with its submission to the journal.

## Registration of research studies

1. Name of the registry: http://www.researchregistry.com.

2. Unique Identifying number or registration ID: researchregistry6066.

3. Hyperlink to your specific registration (must be publicly accessible and will be checked): https://www.researchregistry.com/browse-the-registry#home/6066.

## Guarantor

Dr. Dimitrios Karakitsos, Critical Care Dept., King Saud Medical City, Work address: PO Box 331905, Zip code: 11,373 - Shemaisi, Riyadh, Saudi Arabia, email: karakitsosdimitrios@gmail.com, phone: +966–509816296.
